# Autonomous recognition of erroneous raw key bit bias in quantum key distribution

**DOI:** 10.1038/s41598-025-30095-1

**Published:** 2025-11-29

**Authors:** Matt Young, Marco Lucamarini, Stefano Pirandola

**Affiliations:** 1 Department of Computer Science, University of York, York, YO10 5GH UK; 2 School of Physics, Engineering, and Technology, University of York, York, YO10 5FT UK

**Keywords:** Quantum information, Information theory and computation, Computer science

## Abstract

As quantum key distribution technologies mature, it is pertinent to consider these systems in contexts beyond lab settings, and how these systems may have to operate autonomously. To begin, an abstract definition of a type of error that can occur with regard to the ratio of bit values in the raw key is presented, and how this has an impact on the security and key rate of QKD protocols. A mechanism by which errors of this type can be autonomously recognised is given, along with simulated results. A two part countermeasure that can be autonomously put in place to mitigate against errors of this type is also given. Finally some motivating examples where this type of error could appear in practice are presented to add context, and to illustrate the importance of this work to the development of quantum key distribution technologies.

## Introduction

With the advancement of research into quantum key distribution (QKD)^1,2^, we are moving ever closer to a world in which QKD systems are a part of real-world infrastructure^3,4^. In such a setting, determining if a QKD system is operating as it should is a task that will need to be performed by a QKD system autonomously. In addition to this, downtime of a QKD system should be kept to a minimum, otherwise the operation of critical organisations or infrastructure making use of QKD systems could be severely compromised.

In this work a new scheme is presented to autonomously recognise an error where Bob is no longer able to detect one of the encoding states used by Alice, which manifests as a change in the ratio of bit values in Bob’s raw key. For brevity, in this work this will be referred to as a state detection error, or SD error. SD error recognition is done by performing an additional step using the generated raw key, after which Alice and Bob can automatically put a countermeasure in place allowing the QKD system to continue operation, preventing downtime, and autonomously adding redundancy to QKD systems.

To begin, an introduction of the 3-state BB84 protocol and side-channel attacks are given, which are some of the pre-requisite core concepts used in the main body of this work (Henceforth for the remainder of this work, when discussing the 3-State BB84 protocol in this work, we will state it as such. Any reference to the BB84 protocol is in reference to the original 4-State version of the BB84 protocol). Following this, motivating examples of how an SD error could occur are given in the form of a fault of a measurement device, and of a possible side-channel attack. The consequences of failing to recognise an SD error are discussed, before then going on to present the procedure for recognising an erroneous ratio of raw key bits, after which a countermeasure that can be taken by Alice and Bob is given.

### 3-State BB84

The original QKD protocol proposed by Bennett and Brassard^5^ (BB84) makes use of 4 quantum states to encode information, $|0\rangle$, $|1\rangle$, $|+\rangle$, and $|-\rangle$, where $|\pm \rangle =\frac{1}{\sqrt{2}}(|0\rangle \pm |1\rangle )$. A full description of the BB84 protocol can be found in Appendix A.

There exists a specification and security proof of BB84 that only makes use of three of these states^6^ and this version of the protocol has been demonstrated in practice^7,8^. For illustrative purposes, the three states $|0\rangle$, $|1\rangle$, and $|+\rangle$ will be considered, but any three of the four BB84 states mentioned above can be used. There are only two changes from BB84:

1. When Alice randomly chooses to prepare a state from the phase-basis (also known as the x-basis), she always prepares the $|+\rangle$ state.
2. Only instances from the computational-basis (z-basis) are used for key-generation, whilst all instances from the x-basis, and a small fraction from the z-basis are used for parameter estimation.

It has since been shown by other security proofs that the achievable Secret-Key-Rate (SKR) for the 3-State BB84 protocol is almost identical to that of the BB84 protocol, except for situations with really high attenuation^9,10^. Tamaki et al.^9^ even go on to state that as the two SKRs are so similar, it implies that one of the states in BB84 is redundant. It is this redundancy that this work makes use of to remove the dependency on the encoding state that is no longer being detected by Bob.

### Side-channel attacks

When analysing cyber-security systems to ensure their security, whether they be classical or quantum, ensuring the theoretical principles underpinning these systems are correct and secure is paramount. Within the context of QKD, it is important to ensure that the assumptions on which security proofs of QKD protocols are based are followed. When considering realistic devices, this can be difficult to achieve^11,12^ and this can open up avenues of attack. Such attacks are called Side-Channel attacks^13,14^. Typical examples of more traditional side-channel attacks involve measuring power^15^, electromagnetic emissions^16^, optical emissions^17,18^, or execution time^19^.

An example of a side-channel in QKD that could be leveraged by an attacker to perform a side-channel attack is that of an eavesdropper (henceforth denoted as Eve) having control over measurement devices, where Measurement-Device-Independent (MDI) QKD was proposed^20,21^ in response. Another commonly discussed side-channel is that of multi-photon emission by Alice’s photon source^22,23^. This would allow Eve to take one of the photons for herself and, with the help of a quantum memory, delay measurement of the qubits until after the basis reconciliation stage such that she can make the correct measurement for all of the qubits, for which the decoy-state method was proposed as a countermeasure^24–26^. Eve could also externally interact with the QKD systems, for example, by injecting photons into the system to read information about the state encodings, known as a Trojan Horse attack^27^, or by sending bright pulses of light to Avalanche Photodiode (APD) detectors in order to blind them, before then performing a faked-state attack^28,29^. Another example of a side-channel attack on QKD systems is to measure the other degrees of freedom that are not being used to encode data^30,31^. Information from these measurements can then be used to estimate the prepared state. Work has even been done into considering side-channel attacks against Continuous-Variable (CV) QKD^32–34^. For a more complete account see references^12,35^ and the references therein.

## Motivating examples

Suppose in the passive detection scheme described earlier in this work that there was a complete failure of one of the four detectors. Bob would no longer be able to detect one of the four BB84 states used by Alice. This is an SD error. The SD error recognition scheme presented below would be able to recognise this continued period of there being a fault in the system, and put in place the proposed countermeasure to maintain uptime of the QKD system. This type of prolonged fault could also occur partially, for instance dust could build up in front of one of the detectors acting as an optical attenuator, therefore modifying the distribution mean of the key bits, resulting in a lowering of the entropy of the raw key.

This type of consideration is incredibly important for when QKD implementations move out of labs, and into real-world applications where experts are not immediately available to diagnose hardware faults.

An alternative way SD errors could come about could be due to some adversarial third party. Suppose that some eavesdropper Eve had some side-channel attack capability that allows her to prevent Bob detecting one of the four BB84 states. Approaches that can be used as potential attack vectors have already been investigated in the literature, such as:

- Sending relatively high power laser pulses into either Alice of Bob’s setups in order to damage and/or permanently change the characteristics of their optical components^36,37^.
- Adjusting the incident angle of the light on the input port to Bob’s setup to change the path of the light such that it never reaches one of the detectors^38^.

Another avenue for the introduction of a side-channel attack could be through the intentional introduction by some third party of an additional part to the system that blocks one of the four states on demand, in other words a backdoor, either with or without malicious intent. An important discussion that is currently being considered is that of governments introducing legislation requiring backdoors in systems utilising end-to-end encryption^39,40^. Even though this introduction of a backdoor is not done with malicious intent, it nonetheless provides attackers with a potential new avenue of attack that could be exploited. An alternative scenario could be envisaged, where the manufacturer of some QKD system, manufacturer of a constituent component, or the developer of the software required to operate the system may have malicious intent, and therefore would be in a position to create a side-channel capability of this type for use by themselves or some other party. This may be for personal benefit, or because they have been somehow manipulated by another party into introducing this hidden vulnerability.

As discussed previously, by modifying the ratio of bit values in the raw key, Eve would be reducing the entropy of the raw key, leading to a reduction in the key rate. As such, this side-channel attack is a form of denial-of-service (DoS) attack.

From the perspective of Bob this type of side-channel attack and hardware fault are indistinguishable. Similar to how Bob cannot distinguish between channel noise and eavesdropping by Eve, and must therefore assume that the resulting Quantum Bit Error Rate is produced by an attack, Bob in this case must assume the worst case scenario: if a recognition of an SD error is made, then it is from a side-channel attack.

## Erroneous raw key bit ratio

Prior to presenting the SD error recognition scheme, the type of error itself needs to be specified, as well as assumptions being made about the QKD scheme used to illustrate the results.

For the purposes of this paper it is assumed that the BB84 protocol is being used, where the Z and X bases are both used for key generation and parameter estimation, and are each used 50% of the time. Later on in this work it will be shown how other basis ratios besides 50:50 can be used by this scheme, relaxing this assumption. It will also be assumed that there are four photodetectors that implement a passive detection scheme. Extension of this scheme to active QKD implementations is left for future work.

The type of error considered in this work is as follows: Bob will at some point in the execution of the QKD protocol be unable to receive one of the four states used by the BB84 protocol for the remainder of the QKD session. This could occur in practice in a variety of ways, and these will be discussed in later sections.

An SD error would result in one of the two bases only producing bits of one particular value, lowering the entropy of the raw key. In the context of the assumed BB84 protocol, this would change the ratio of key bits from 2 : 2, to 1 : 2 or 2 : 1 depending on which state is no longer being received by Bob. Later in this work it will be useful to think about the average bit value of the raw key, which for the above ratios are 1/2, 2/3, and 1/3 respectively.

To illustrate why the lowering of the entropy of the raw key is not desirable, consider the Devetak-Winter rate^41^ for the SKR $R=I(A:B)-I(A:E)$. Assuming an absolute best-case scenario, the mutual information *I*(*A* : *E*) can be ignored as it only detracts from the secret key rate. *I*(*A* : *B*) can be replaced by it’s definition $I(A:B)=H(A)-H(A|B)$ and again, assuming the best-case scenario, the conditional entropy *H*(*A*|*B*) becomes zero (ie. Alice and Bob’s raw keys perfectly match). As such, the entropy of Alice’s raw key *H*(*A*) can be taken to be an upper bound on Alice and Bob’s mutual information, and by extension, the secret key rate:

1 $$\begin{aligned} R=I(A:B)-I(A:E)\le I(A:B)\le H(A) \end{aligned}$$

Therefore if the entropy of the raw key is reduced, the maximum possible secret key rate is also reduced.

## Error recognition

In order for Alice and Bob to take action against an SD error, they must first be able to recognise it. Alice could by means of classical communication tell Bob how many qubits were transmitted, and then Bob could calculate the loss using the number of qubits he received. However this loss may have several causes, one of which could be an SD error, but it could also be due to an increase in the channel noise. This approach doesn’t yield sufficient information for Bob to make a determination as to the presence of an SD error. As can be seen by the Binary Shannon Entropy:

2 $$\begin{aligned} H_2(p)=-p\log _2(p)-(1-p)\log _2(1-p) \end{aligned}$$

entropy is dependent on the probability distribution of the 0 and 1 bits in the raw key, where $p=P(X=0)$ with *X* being a binary random variable representing Bob’s measurement outcomes. As an SD error decreases the entropy of the raw key, but general channel losses do not, estimating this probability distribution will allow Bob to make a determination as to the presence of the SD error.

If Bob were to sample the raw key bits live as they were obtained and calculated the mean of these values, he would see his calculated estimate of the mean deviate significantly from the nominal mean for the protocol. For example, using the assumed BB84 protocol as stated above, the nominal mean is 0.5. It is important to note that whilst an SD error causes a change in the mean raw key bit value, SD errors may not be the only possible causes of a biased raw key mean value. In this work we limit the scope only to SD errors being the cause of a biased raw key bit mean value, however future work could look to further address this assumption. This problem is analogous to determining the bias of a biased coin, or more formally, determining the parameter of a random Bernoulli variable.

However, in order to do this, two questions must now be answered:

1. How many samples must Bob take in order to calculate a useful estimate of the mean?
2. What does it mean for the estimated mean to deviate significantly?

This first question is considered first, and by doing so will lead to a natural solution to the second question.

First, the actual mean of the distribution is defined as $\mu$, and Alice and Bob’s estimate of the mean as:

3 $$\begin{aligned} \hat{\mu }=\frac{1}{n}\sum _{i=0}^nR_i \end{aligned}$$

where *n* is the number of key bits sampled to calculate the estimate of the mean, and $R_i$ is the *i*-th bit sampled from the raw key. The difference between the actual value and the estimated mean can then be quantified as $|\mu -\hat{\mu }|\le \delta _\mu$ where $\delta _\mu >0$ is a parameter that specifies how close the estimate of the mean should be to the actual value, or rather how precise the estimate is.

However, as $\hat{\mu }$ is calculated using samples from a random variable, there is no guarantee that the above condition will hold. As such, the following can be specified:

4 $$\begin{aligned} P(|\mu -\hat{\mu }|\le \delta _\mu )\le 1-{p_{FA}},\quad 0\le {p_{FA}}\le 1 \end{aligned}$$

where $p_{FA}$ is another parameter specifying that the condition on the precision of the estimate $\hat{\mu }$ holds with probability $1-{p_{FA}}$. The parameter $p_{FA}$ can also be interpreted as the probability that this scheme produces a false alarm. It is worth noting that for a block size of $n_b$ bits, the number of expected false alarms would be $n_{FA}=(n_b-n+1)*p_{FA}$, where *n* is the number of bits used to estimate the mean. As such, in order to avoid a false alarm, choices of $n_b$ and $p_{FA}$ should be made such that $n_b<\frac{1}{p_{FA}}+n-1$.

The inequality given in Eq. (4) has been widely studied in the field of inferential statistics. The Chernoff-Hoeffding bound provides a lower bound on the number of samples required^42^:

5 $$\begin{aligned} n\ge \frac{ln(2/{p_{FA}})}{2{\delta _\mu }^2}. \end{aligned}$$

**Fig. 1:**
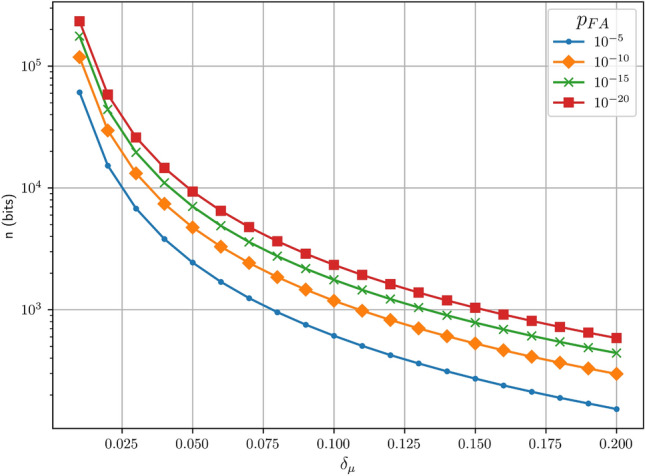
Logarithmic-scale plot of the number of bits from the Chernoff-Hoeffding bound against $\delta _\mu$ for different values of ${p_{FA}}$.

As can be seen from Fig. 1, the number of samples required is inversely proportional to both $\delta _\mu$ and ${p_{FA}}$, however $\delta _\mu$ scales particularly poorly. These *n* samples used are obtained from the latest *n* bits received by Bob, and so this operation can be considered to be a sliding window over the raw key with a window size of *n* bits.

Next is to consider the problem of finding some threshold after which it can be considered that the estimate of the mean has deviated significantly. From here on this threshold shall be referred to as the recognition threshold and shall denoted by *t*. The nominal mean shall be denoted by $\mu _N$. By doing this, other values of the nominal mean are allowed besides the 1/2 used in this work for illustrative purposes. An example where a nominal mean besides 1/2 arises is a variation of the BB84 protocol^43^ that does not use an equal proportion of bits from the Z and X bases, but rather uses some proportion *p* of bits from the X basis such that $0<p\le 0.5$. Then, by definition of $\delta _\mu$ it can be said that under nominal conditions $\mu _N-\delta _\mu \le \hat{\mu }\le \mu _N+\delta _\mu$. If the recognition threshold were to be set to less than $\delta _\mu$ then due to statistical fluctuations, there would be false alarms outside of the specification provided by the parameters $\delta _\mu$ and ${p_{FA}}$, even under nominal conditions. Therefore this imposes the bound of $t\ge \delta _\mu$. As such, the closest possible recognition threshold can be considered to be $t=\delta _\mu$.

**Fig. 2:**
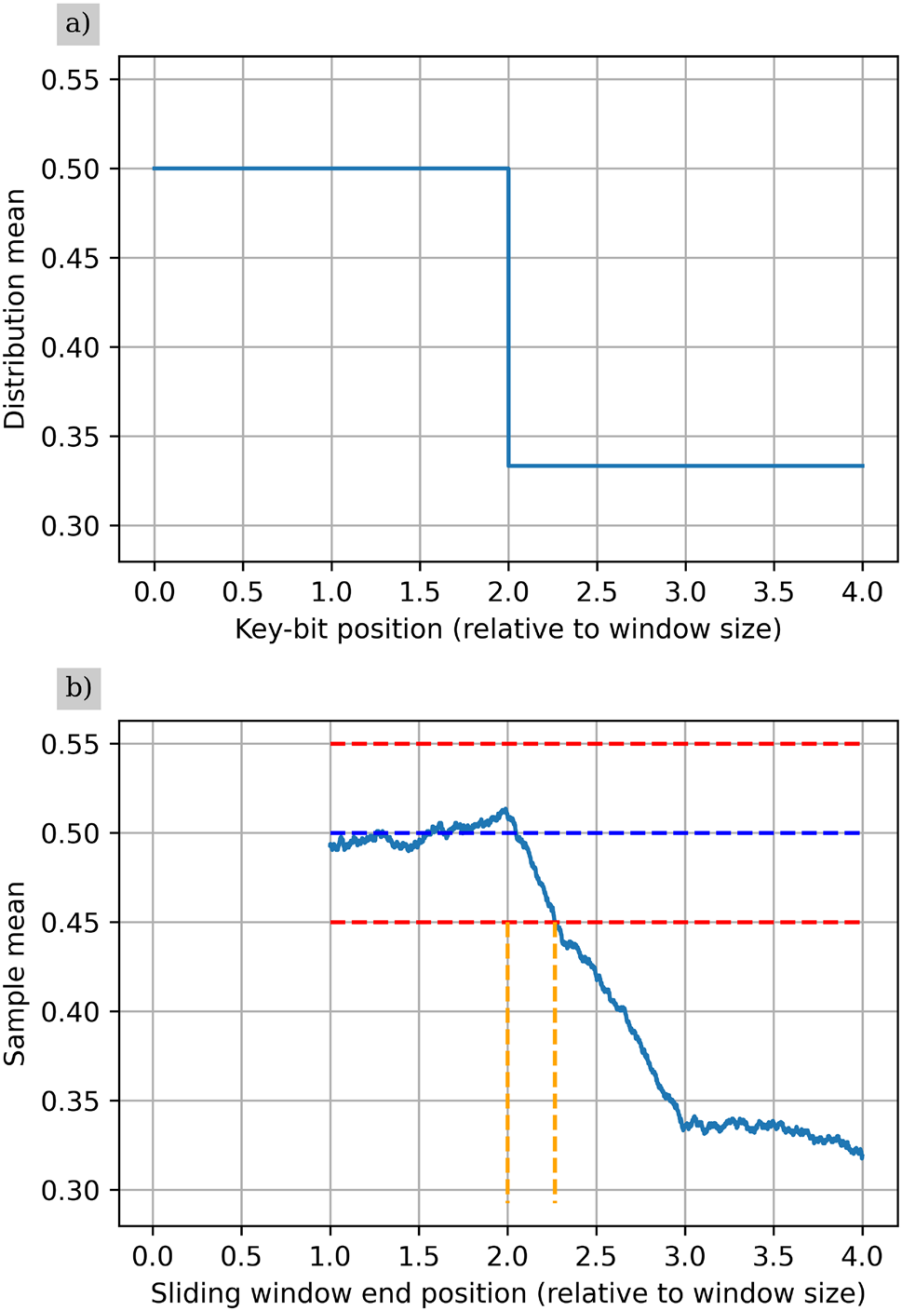
(**a**) The probability distribution used to generate the raw key bits. (**b**) Bob’s estimated mean over time. The blue dashed line represents the nominal mean value, and the red dashed lines represent the upper and lower recognition thresholds. The first and second vertical orange dashed lines show where the SD error occurred, and where the recognition of the SD error was made respectively. This simulation was done with parameters $\delta _\mu =0.05$ and $p_{FA}=0.001$, hence a value for *t* of $t=0.05$ was chosen as can be seen by the recognition thresholds.

A simulation of an SD error and recognition of it^44^ is shown in Fig. 2. The simulation draws key bits from a uniform probability distribution for a given number of raw key bits, after which the probability distribution is changed to reflect the failure of one of the four detectors. In the case of Fig. 2 this corresponds to the failure of one of the detectors that produce raw key bit values of 1. The sample mean then begins to change, and crosses the lower recognition threshold at a sample mean of 0.45.

It is important to note that the Chernoff-Hoeffding bound requires that the samples used to estimate the mean are independent. It is a typical assumption in QKD that the states that Alice encodes into her qubits are uniformly random and independent of one another, and therefore under this assumption, the requirement of the Chernoff-Hoeffding bound that the samples be independent is satisfied.

Once a fault has occurred the sample mean can be modelled by taking samples from two different binomial distributions, which can be rewritten as:

6 $$\begin{aligned} \hat{\mu }=\frac{1}{n}{(\sum ^{n_0-1}_{i=0}x_{0i}+\sum ^{n_1-1}_{i=0}x_{1i})} \end{aligned}$$

where $x_{ji}$ is the *i*-th sample from the *j*-th probability distribution, $n=n_0 + n_1$ , and the probability distributions labelled 0 and 1 are the probability distributions when there isn’t an SD error and when there is an SD error respectively.

This difference means that $\hat{\mu }$ will not truly reflect the mean of any one of the distributions, but rather present a weighted average based on the number of samples from each, given by

7 $$\begin{aligned} \hat{\mu }=\frac{1}{n}\sum _{i=0}^{k-1}\mu _in_i~. \end{aligned}$$

This means that the estimate of the mean will take time to move away from that of $\mu _N$ once the SD error has occurred. This can be seen in Fig. 2 by the sloped line once the probability distribution of raw key bits changes. How far away from $\mu _N$ is tolerable can be bounded by $\mu _N-t\le \hat{\mu }\le \mu _N+t$. This is illustrated in Fig. 2 by the red dashed lines representing the upper and lower recognition thresholds.

## Countermeasure

Due to an SD error, there will be a region of the raw key that is partially insecure. A two part countermeasure can be put in place. The first part is to discard raw key bits that form the partially insecure region of the raw key. The second part is to switch the protocol being used from the BB84 protocol to the 3-state BB84 protocol, which removes the dependence on the fourth state that is now no longer being received, and restores the raw key to having maximal entropy.

### Discarding compromised key bits

In order to discard the required raw key bits, how many bits should be discarded needs to be quantified first. First, the difference between the nominal mean $\mu _N$, and the current estimate of the mean $\hat{\mu }$ can be expressed. Specifically the case where this difference first exceeds the recognition threshold *t* needs to be considered, and is given by $|\mu _N-\hat{\mu }|=t$. It is also useful to express $\hat{\mu }$ in the form:

8 $$\begin{aligned} {\hat{\mu } = \mu _0 + (\mu _1 - \mu _0)\frac{k}{n}} \end{aligned}$$

where *k* is the number of samples taken after the occurrence of a fault, with a total number of samples *n*. Considering that recognition of a fault occurs when $|\mu _N-\hat{\mu }|=t$, then the expected number of samples required for recognition of a fault is given by:

9 $$\begin{aligned} {k^\star = \frac{t}{|\mu _1 - \mu _0|}\,n}. \end{aligned}$$

By using specific values of $\mu _0=0.5$, $\mu _1=0.3\dot{3}$, and $t=0.05$ from the scenario presented in Fig. 2, it is seen that $k^\star =0.3n$.

Hence, when Bob recognises an SD error, he could discard 0.3*n* bits worth of the raw key, however as this is only the case on average, sometimes he would not discard enough, and there would still be portions of insecure key remaining. A naive solution to this would be to increase this value of 0.3*n* by some arbitrarily chosen multiplicative constant, large enough to discard enough of the raw key the vast majority of the time. However instead of an arbitrary constant a bit more of a sophisticated choice can be made.

For this, Hoeffding’s inequality^45^ will be used:

10 $$\begin{aligned} {\Pr \!\left( |\hat{\mu }-\mathbb {E}\hat{\mu }|\ge \delta \right) \le 2e^{-2n\delta ^2}.} \end{aligned}$$

Recognition of a fault is guaranteed once $|\mu _N-\mathbb {E}\hat{\mu }(k)|\ge t+\delta$, which implies, with probability at least $1-2e^{-2n\delta ^2}$,

11 $$\begin{aligned} {k \ge \frac{t+\delta }{|\mu _1-\mu _0|}\,n .} \end{aligned}$$

For some failure probability $\varepsilon _D$, by letting $1-\varepsilon _D=1-2e^{-2n\delta ^2}$ a choice of $\delta =\sqrt{\tfrac{1}{2n}\ln \!\tfrac{2}{\varepsilon _D}}$ can be made, which gives:

12 $$\begin{aligned} {k \ge \frac{t}{|\mu _1-\mu _0|}\,n + \frac{1}{|\mu _1-\mu _0|}\sqrt{\frac{n}{2}\ln \!\frac{2}{\varepsilon _D}}} \end{aligned}$$

and as such, the number of key bits to discard can be set to:

13 $$\begin{aligned} {n_{\textrm{discard}} = \Bigg \lceil \frac{t}{|\mu _1-\mu _0|}\,n + \frac{1}{|\mu _1-\mu _0|}\sqrt{\frac{n}{2}\ln \!\frac{2}{\varepsilon _D}} \Bigg \rceil ,} \end{aligned}$$

where $\varepsilon _D$ is included in the overall composable security parameter $\varepsilon _{\textrm{Sec}}$. It is important to note that due to the use of Hoeffding’s Inequality which is an upper bound, this result itself is also an upper bound.

The failure probability $\varepsilon _D$ is the probability that not enough bits have been discarded, and is related to the number of standard deviations used which shall be denoted as *z*. This relationship is given by:

14 $$\begin{aligned} {\text {erf}\left( \frac{z}{\sqrt{2}}\right) =1-\varepsilon _D} \end{aligned}$$

where *erf* is the error function. However it is more useful for the number of standard deviations *z* to be the subject of the expression, such that we can use a specified value for the security parameter $\varepsilon _D$ to calculate the required number of standard deviations to use. This gives:

15 $$\begin{aligned} z=\sqrt{2}\times \text {erf}^{-1}(1-\varepsilon _D) \end{aligned}$$

Using Eq. (14), it is possible to set $z=1$, find the corresponding value of $\varepsilon _D$, and use this value in the second term of Eq. (12) to find one standard deviations worth of bits as a function of *n*. In this case, there is a square root relationship between the number of bits in one standard deviation and *n*.

**Fig. 3:**
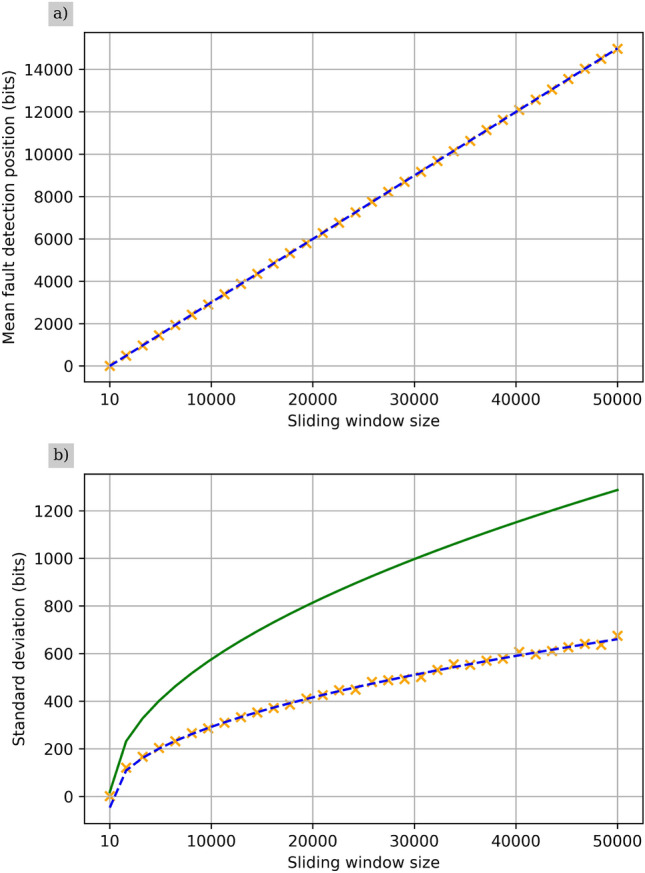
(**a**) This plot shows how the number of insecure raw key bits scales with the size of the sliding window. The dashed line shows the linear regression model that was fit to the dataset obtained via simulation. (**b**) This plot shows the standard deviation of the results from the simulation. It was observed that this data exhibits a square-root scaling, and so a square-root regression model was fit to this data. The dashed line shows this regression model. The solid green line is the analytic upper bound on this quantity, as obtained from the second term in Eq. (12), using a value of $\varepsilon _D$ corresponding to 1 standard deviation obtained using Eq. (14). For these simulations, the same parameters as with Fig. 2 were used, and 2500 simulations were run for each window size.

In order to reinforce these analytical results, simulations were run for different values of the sliding window size. For each of these different values, many SD errors were simulated, and the time when each SD error was recognised was recorded. For the case of the simulated results in Fig. 3 2500 SD errors were simulated per data-point. The mean number of samples required for recognition ($n_r$), and the standard deviation were calculated. Figure 3 shows plots for these two quantities where linear and square root relationships were found for $n_r$ and the standard deviation respectively.

A linear regression was performed for the mean SD error recognition position in the sampling window, and a linear regression model of $n_r=\alpha n-\beta$ was found, with $\alpha = 0.300$, $\beta = 5.058$, and a root mean squared error (RMSE) of 9.156 bits. This model scales linearly with a scaling factor incredibly close to the value of 0.3 as expected through the earlier use above of Eq. (9).

For the standard deviation it was noticed that it exhibited square root scaling behaviour, and so a square root regression model of $\sigma =\sqrt{\alpha n-\beta }$ was fit to this data, this time with $\alpha =8.770$, $\beta =2332.743$, and a RMSE of 11.068 bits. The square root relationship between the number of samples *n* and the standard deviation observed in these results matches the results obtained analytically earlier in this section.

As an example, for a sampling window size of 50,000 bits, using the regression models, the average error recognition position is 14,993 bits, and the standard deviation is 661 bits (These quantities were rounded up to the next whole bit). Choosing a value of $\varepsilon _D=10^{-10}$ which is a typical choice for QKD applications and using Eq. (15), we require 6.467 standard deviations. This would result in $\lceil 14993+(6.467\times 661)\rceil =19268$ bits needing to be discarded.

Now that the number of raw key bits to discard can be calculated, it is important to consider how discarding these affects the key rate. If the length of the raw key is taken to be $l_r$ and the number of discarded raw key bits to be $n_d$, then the proportion of remaining raw key bits can be calculated, and the key rate *R* is given by:

16 $$\begin{aligned} R'=\frac{(n_4-n_d)}{l_r}R_4+\frac{n_3}{l_r}R_3 \end{aligned}$$

Here $R_4$ and $R_3$ are the key rates for the BB84 and 3-state BB84 protocols respectively, and likewise $n_4$ and $n_3$ are the number of raw key bits distributed by each of the protocols, with $n_4$ being the number of raw key bits distributed prior to the discarding of raw key bits. We do this as the key rates may not necessarily be the same.

However as discussed earlier, for cases except those with high channel attenuation, the key rates have been shown in the literature to be almost identical^9,10^. As such by setting the two key rates $R_4$ and $R_3$ to be the same, Eq. (16) reduces down to: $R'=\frac{l_r-n_d}{l_r}R$.

### Transitioning to 3-State BB84

Once an SD error is recognised, it is known that one of the states can no longer be used. As the BB84 protocol requires use of all four states, the naive approach would be to terminate the QKD session. Whilst this would be a secure response to recognition of the SD error, this could cause downtime of potentially critical infrastructure. It would be ideal if this could be avoided.

A more sophisticated approach would be to change the QKD protocol from BB84 to 3-State BB84, where the missing state in the 3-State BB84 specification corresponds to the state no longer being detected by Bob. However Alice and Bob cannot just switch between protocols as their behaviour differs, albeit only slightly. There are four required changes that need to be made.

The first requirement is for Bob to make Alice aware that she needs to switch to 3-State BB84, and which state needs to be removed. On the face of it, revealing this information over the classical channel might seem like it presents a security concern. However, in accordance with Kerckhoff’s principle^46^, all parties including some eavesdropper Eve know the specifications of any ongoing protocol. Eve does not gain any more information than she would normally have if Alice and Bob were using 3-State BB84 from the outset.

The second requirement is that Alice needs to change her state generation procedure to that of 3-State BB84.

The third requirement is that Alice needs to change the instances used for parameter estimation to that of 3-State BB84. It is worth noting that Alice does all of these changes, and Bob does not need to change anything about his part of the protocol.

The fourth requirement is that Alice and Bob end the block of BB84 raw key bits after discarding the compromised bits, and begin a new block for the 3-state BB84 protocol. This is to avoid a clash between any potential differences with regards to how the security proofs of either protocol handle the later classical post-processing stages.

Alice and Bob can now continue to perform QKD and the uptime of the system has been maintained.

## Conclusion

This work began by defining a type of error, for which an autonomous error recognition scheme was devised using binomial parameter estimation to detect an erroneous bias in the raw key bits. Simulations were developed to show the operation of the recognition scheme, and certain characteristics of the scheme were investigated, such as how long it takes to recognise an error once it has begun.

A two-stage countermeasure was proposed that allows for continued operation of the QKD system after recognition of an error. The first stage required discarding raw key bits that were generated after the error began, but before the error was recognised. The second stage involves switching from BB84 to the 3-State BB84 protocol to remove the dependence on the state that is no longer being detected by Bob, allowing for a secure key to once again be generated.

Finally, there was discussion on how this error recognition scheme could be useful for applications such as fault detection of QKD implementation hardware, or a hypothetical type of side-channel attack.

Future work may look to further investigate different components within QKD implementations, how errors with those components result in changes within the results observed by Bob, and how autonomous recognition of these other types of errors could be done. This relates to the observation made earlier in the paper that SD errors may not be the only possible cause of a biased raw key bit mean value. Future work could investigate if there are other causes of a biased raw key bit mean value and potential methods of identifying the different causes. Future work could also consider that the Chernoff-Hoeffding bound and Hoeffding’s Inequality used in this work are upper bounds, and not exact solutions which would allow for faster error recognition. The analysis code produced for the simulations could also be applied to a real-world dataset gathered from experimental implementation to further validate the efficacy of the presented error recognition scheme. Future work could also look at extending the scenario presented in this paper to consider how an additional detector fault could be handled, for instance by transitioning from the 3-state BB84 protocol to the B92 protocol^47^ in the case of having two non-orthogonal states remaining. Additionally, as discussed earlier, in order to prevent a false alarm of a SD error, choices of the block size $n_b$ and probability of false alarm $p_{FA}$ should be chosen such that $n_b<\frac{1}{p_{FA}}+n-1$. Future work could investigate implementing periodic re-testing once a recognition of a SD error is made, such that if it was a false alarm, normal 4-state BB84 operation can be resumed. Finally, it is important to note that a composable security proof of the scheme described in this work is not provided. In this work we discuss some aspects of security using the security parameter formalism when discussing the parameter $\varepsilon _D$, which is the formalism used when conducting such proofs, and as such we provide some of the groundwork for such a proof to be completed in future work.

## Supplementary Information

Supplementary Information.

## Data Availability

Simulation code that supported the findings of the work has been deposited in a Zenodo repository, accessible at: https://doi.org/10.5281/zenodo.13738091. This repository is also referenced within the manuscript.
