# Supplementary material for: Autonomous recognition of erroneous raw key bit bias in quantum key distribution
Source: Sci Rep. 2025 Nov 29;16:598. doi: 10.1038/s41598-025-30095-1 (PMC12775369; doi:10.1038/s41598-025-30095-1)
Supplement: Supplementary file 1 — Supplementary Information. [file 41598_2025_30095_MOESM1_ESM.pdf]

A BB84 Protocol

The BB84 QKD protocol uses two Mutually Unbiased Bases (MUBs). In most cases, these are the Computational and Phase Bases (otherwise known as the Z basis and X basis respectively). If two bases are Mutually Unbiased Bases, then any basis state from one basis, measured using the other basis will give a uniform probability distribution of measurement outcomes:

$$|\langle\psi_Z|\psi_X\rangle|^2 = |\langle\psi_X|\psi_Z\rangle|^2 = \frac{1}{2} \tag{1}$$

where ψ_Z and ψ_X are either of the basis vectors from the Z and X bases respectively.

The first step of an iteration of the BB84 protocol is for Alice to generate a uniformly random key bit k_i and choice of basis b_i. Then, Alice encodes this in a quantum state $|\psi_i\rangle$ using the below encoding scheme:

$$|\psi_i\rangle = \begin{cases} |0\rangle, & k_i = 0, b_i = 0 \\ |1\rangle, & k_i = 1, b_i = 0 \\ |+\rangle, & k_i = 0, b_i = 1 \\ |-\rangle, & k_i = 1, b_i = 1 \end{cases} \tag{2}$$

She then transmits this qubit to Bob over some quantum channel.

When Bob receives the qubit, he makes a measurement in a random choice of either the Z or X bases. Bob uses the following scheme to decode the measured results into a raw key bit r_i, where o_i is Bob's measurement outcome:

$$r_i = \begin{cases} 0, & o_i = |0\rangle \text{ or } |+\rangle \\ 1, & o_i = |1\rangle \text{ or } |-\rangle \end{cases} \tag{3}$$

They then repeat this procedure how ever many times are needed to generate twice the desired number of key bits. Once Bob has made measurements on all of the qubits, he announces such to Alice over the classical channel. Alice then announces over the classical channel which bases she used to encode each qubit. Bob announces for which qubits he made the incorrect choice of basis, and both Alice and Bob discard those results. This step is called Basis Reconciliation or Sifting, and on average, this leaves half the classical bits remaining. These remaining n classical bits form the encryption key to be used in a One-Time Pad scheme between Alice and Bob.

Table 1 below gives an example of 8 bits being distributed by Alice under the BB84 protocol.

Alice's Key Bits (K)	0	1	1	0	1	0	0	0
Alice's Basis Bits (B)	0	1	0	0	1	1	1	0
Alice's Basis Choices	Z	X	Z	Z	X	X	X	Z
Alice's Encoded Qubits	$\|0\rangle$	$\|-\rangle$	$\|1\rangle$	$\|0\rangle$	$\|-\rangle$	$\|+\rangle$	$\|+\rangle$	$\|0\rangle$
Bob's Basis Choices	X	Z	Z	Z	Z	X	X	X
Bob's Measurements	-	0	1	0	1	+	+	1
Bob's Key Bits (R)	1	0	1	0	1	0	0	1
Alice's Announced Bases	Z	X	Z	Z	X	X	X	Z
Bob's Incorrect Basis Choices	•	•			•			•
Alice and Bob's Final Key			1	0		0	0	

Table 1. An example of 8-bits being distributed using the BB84 Quantum Key Distribution protocol.
